# Synthesis, Characterization, and Osteogenic Ability of Fibrillar Polycaprolactone Scaffolds Containing Hydroxyapatite Nanoparticles

**DOI:** 10.1021/acsami.4c20796

**Published:** 2025-03-31

**Authors:** Taisa N. Pansani, Carlos Alberto de Souza Costa, Lais M. Cardoso, Amanda M. Claro, Hernane da Silva Barud, Fernanda G. Basso

**Affiliations:** † Department of Dental Materials and Prosthodontics, São Paulo State University (UNESP), Araraquara School of Dentistry, Araraquara 14801-903, Brazil; ‡ Department of Physiology and Pathology, São Paulo State University (UNESP), Araraquara School of Dentistry, Araraquara 14801-903, Brazil; § Biopolymers and Biomaterials Laboratory (BioPolMat), University of Araraquara (UNIARA), Araraquara 14801-340, Brazil

**Keywords:** scaffolds, hydroxyapatite, osteogenesis, osteoblasts, biocompatible materials

## Abstract

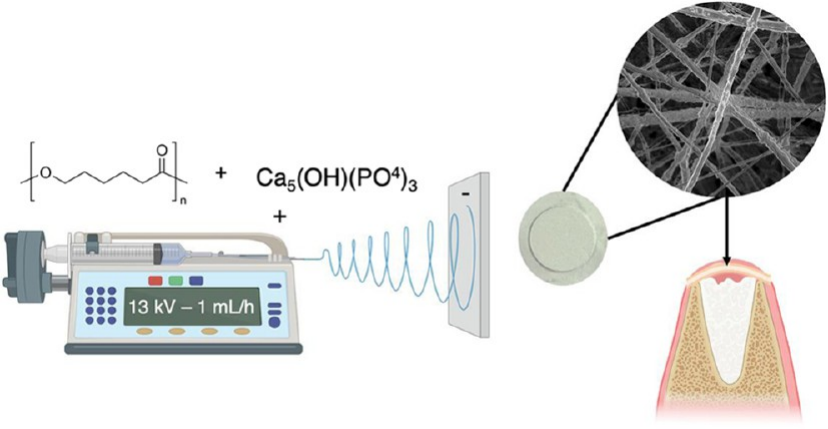

Polymer-based scaffolds for bone regeneration aim to mimic the structure and function of the collagen-rich extracellular matrix. Hydroxyapatite incorporated into these biomaterials improves their mechanical and biological properties due to its bioactive osteoconductive nature. The objectives of this study are to synthesize and characterize polycaprolactone (PCL) scaffolds containing hydroxyapatite nanoparticles (HAn) at 1, 2.5, 5, and 7% concentrations and to determine their cytocompatibility and osteogenic potential. Fiber thickness (*n* = 240) and interfibrillar space (*n* = 8) of PCL scaffolds were characterized by scanning electron microscopy (SEM). The PCL scaffolds were evaluated concerning their thermal degradation (TGA), calcium release, and hydrophilicity (WCA). Preosteoblasts were seeded on PCL scaffolds and assessed regarding their viability (AlamarBlue, *n* = 8), collagen synthesis (SR, *n* = 8), total protein synthesis (TP, *n* = 8), alkaline phosphatase activity (ALP, *n* = 8), deposition of mineralization nodules (MN, *n* = 8), and cell adhesion (fluorescence microscopy). The data analyses of the biomaterials, including TGA, energy dispersive spectroscopy (EDS), and Fourier transform infrared spectroscopy (FTIR), were interpreted descriptively. The quantitative data were statistically analyzed (α = 5%). Scaffolds without HAn exhibited thicker fibers. The higher incorporation of HAn in the PCL scaffolds increased the interfibrillar spaces and resulted in greater P and Ca peaks (*p* < 0.05), as well as broader peaks representing the P–O group (FTIR). TGA demonstrated that PCL scaffold degradation was inversely proportional to their HAn concentration. Higher percentages of cell viability were observed with the incorporation of HAn. ALP activity increased in cells seeded onto PCL scaffolds containing 2.5% HAn. Deposition of MN was directly proportional to the amount of HAn incorporated. HAn incorporated into PCL scaffolds interferes with the physicochemical properties of these biomaterials and favors in vitro osteogenesis.

## 1 Introduction

The remarkable progress reached over the last decades in the field of tissue engineering has motivated researchers to develop biomaterials capable of mimicking the structure and function of the collagen-rich extracellular matrix, serving as support for the adhesion, proliferation, and differentiation of cells.^1^ Ideally, scaffolds for bone regeneration must be biocompatible, biodegradable, and osteoconductive. These biomaterials should exhibit porous structures to allow internal cell migration, proliferation, and synthesis of the extracellular matrix and present a modulus of elasticity similar to that of bones.^2^

Scaffolds obtained by the electrospinning technique have been extensively assessed for bone regeneration because of their uniform fiber distribution. These biomaterials allow the incorporation of molecules capable of improving their bioactivity and mechanical properties.^3,4^ Polycaprolactone (PCL) is a biocompatible and biodegradable low-cost synthetic polymer that simulates the structure of the extracellular bone matrix.^5^ Additionally, electrospun fibrillar scaffolds (EFS) resemble collagen fibers of the bone extracellular matrix, turning them into excellent biomaterials to be used for inducing bone-guided regeneration.^6^ However, due to the fact that EFS are bioinert, do not allow high formation of calcium phosphate, and exhibit slow degradation, these biomaterials require incorporation of bioactive and osteoinductive molecules.^7^

Hydroxyapatite (HA) is an endogenous calcium phosphate that induces bone neoformation and mineralization. HA, a bioactive material with osteoinductive potential, has been used as a supporting material for filling bone defects, since it can simulate the inorganic phase of mineralized tissues.^8^ In previous studies, Hassan and Sultana in 2017^9^ used the electrospinning technique to characterize the physicochemical properties of polymeric nanofibers prepared with PCL containing hydroxyapatite nanoparticles (HAn). It is known that HAn increases the proliferation of osteoblasts, the activity of the alkaline phosphatase enzyme, and the concentration of calcium in the extracellular matrix.^10^ Such inorganic particles at the nanometer scale improve the osteogenic differentiation and expression of genes related to mineralization compared to the same particles at the micrometer scale.^11−13^

The use of hydroxyapatite (HAn) alone as a biomaterial already demonstrates remarkable osteogenic potential, as it closely mimics the mineral component of natural bones and provides a conducive environment for cell attachment, proliferation, and differentiation.^14^ Combining HAn with polycaprolactone (PCL), we can have a material that is not only biologically effective but also highly cost-efficient, which is a significant advantage for broader applications and accessibility.^15^ Moreover, the literature reveals significant variability in the parameters employed for scaffold fabrication, including the choice of solvents, their concentrations, and the proportion of hydroxyapatite nanoparticles (HAn). This inconsistency can introduce substantial differences in the results, highlighting the need for standardized methodologies to ensure reliable and comparable outcomes.^16^

Based on these scientific data, one may suggest that the incorporation of different concentrations of HAn in PCL nanofiber scaffolds would improve the osteoinductive potential of these polymeric materials. However, it seems mandatory to previously evaluate the physical, mechanical, and biological properties of these biomaterials as well as to determine their cytocompatibility and osteogenic potential when applied in direct contact with a primary culture of osteoblasts. The hypothesis of this study is that the incorporation of HAn into the fibers of PCL fibrillar scaffolds can stimulate preosteoblastic cells, favoring the process of mineralized matrix deposition and bone neoformation.

## 2 Experimental Section

### 2.1 Scaffolds’ Fabrication

**Figure 1:**
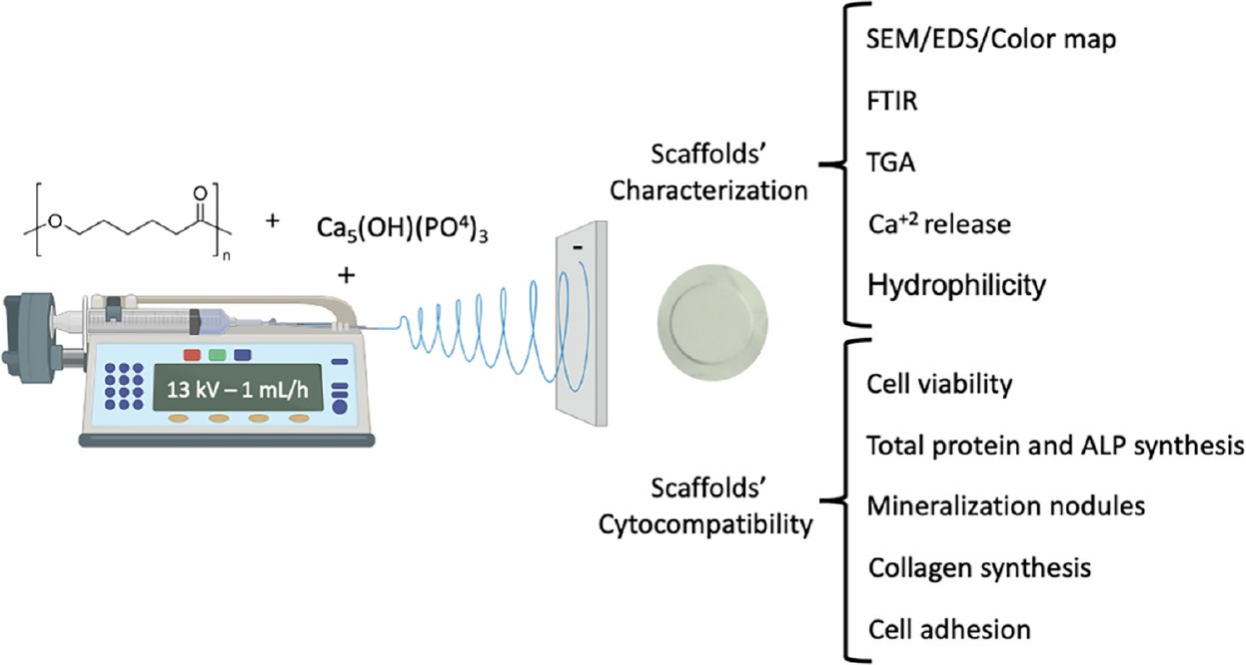
Schematic diagram of the experimental approach and research objectives. Made using BioRender software.

Poly(ε-caprolactone) scaffolds (PCL; 10% w/v; Sigma-Aldrich, Saint Louis, MO) were prepared using the electrospinning technique. For this purpose, concentrations of 1, 2.5, 5, and 7% (w/v in relation to PCL solution) of hydroxyapatite nanoparticles (HAn; <200 nm; Sigma-Aldrich) were added to chloroform/dimethylformamide solutions (8:2 v/v) containing 10% PCL under stirring for 24 h at room temperature. Pristine PCL scaffolds were used as controls. Then, the solutions were transferred to a 5 mL syringe with a stainless steel needle, coupled to an automatic injection pump (DKScientific, Holliston, MA) at a flow rate of 1 mL/h. The parameters used were as follows: voltage of 13 kV and distance between the needle and the collector of 15 cm. After the scaffolds were fabricated, structural characterization and cytocompatibility analyses were conducted, as illustrated in the schematic diagram in Figure 1.

### 2.2 Structural Characterization of Scaffolds by SEM, EDS, and Elemental Color Map Analysis

After the synthesis of the scaffolds, they were dried in a desiccator (room temperature) for 72 h to evaporate the solvent. Then, a sterilized dermatological punch was used to obtain round-shaped samples of 3 mm in diameter, which were gold-sputtered and then evaluated in a scanning electron microscope (SEM; Inspect Scanning Electron Microscope-S50, FEI, Hillsboro, OR) attached to the energy dispersive spectroscopy (EDS) system for structural characterization and analysis of the incorporation of HAn into the nanofibers.

To examine the structure of the fibers of the scaffolds in greater detail, images were captured at 20,000× magnification using a high-resolution scanning electron microscope (FEI Magellan 400 L, FEI, Hillsboro, OR). Additionally, an elemental color map analysis (SEM, Philips XL-30 FEG, Bruker, EUA) was conducted to determine the distribution of the chemical elements present in the scaffolds.

To measure the diameter of the fibers, two images were obtained from different areas of each sample at a magnification of 2000× (*n* = 8/group). Then, the diameter of 240 fibers (30 per image) and the proportion of interfibrillar spaces (%) were digitally evaluated using ImageJ software (National Institute of Health-NIH, Bethesda, MD).

### 2.3 Characterization of Nanofibers by Fourier Transform Infrared Spectroscopy (ATR-FTIR)

The absorption spectra in the infrared region were recorded in reflection geometry by an ATR-FTIR spectrometer (PerkinElmer Inc., model Spectrum 100, Massachusetts). The chemical characterization of the functional groups present in the nanofibers was determined from the analysis of the absorption spectra of the samples compared to infrared spectroscopic information. The samples were prepared, and the wavenumber reading range was from 4000 to 650 cm^–1^.

### 2.4 Thermogravimetric Analysis (TGA)

Dynamic thermogravimetric measurements of PCL nanofibers containing different concentrations of HAn were performed using a TGA/DSC system instrument (model SDT Q600, New Castle, DE). In this study, 5 mg of specimens (nanofibers) were submitted to heat in the temperature range of 30–600 °C, with a heating rate of 10 °C/min under a nitrogen atmosphere.^17^ The thermal stability of PCL, HAn, and PCL loaded with HAn was analyzed by the obtained mass loss curves, and the residue content of samples was determined.

### 2.5 Calcium Release Analysis

To evaluate the calcium release of nanofibers containing HAn, the samples were submerged in 600 μL of ultrapure water for periods of 1, 3, 7, 14, and 21 days. The collected extract was kept at a temperature of −20 °C until the moment of the test. This evaluation was performed using the Calcio Liquiform Kit end-point assay (Labtest Diagnóstico S.A., Lagoa Santa, MG, Brazil) following the manufacturer’s recommendations. This assay analyzes the reaction of supernatant calcium with phthalein purple in an alkaline medium, forming a violet complex. After the reaction, the amount of calcium was evaluated by means of a spectrophotometer at 570 nm (Synergy H1 Hybrid Multimode Microplate Reader, Biotek, Winooski, VT) with the aid of a predetermined standard curve.

### 2.6 Hydrophilicity Analysis (Water Contact Angle (WCA))

The surface property of the PCL fibers containing different concentrations of HAn was characterized with a measuring system DataPhysics OCA 15 (DataPhysics Instruments GmbH, Filderstadt, Germany). The scaffolds were prepared at 13 mm diameter glass coverslips (Perfecta, São Paulo, SP, Brazil), and 2 μL of a deionized water droplet was dropped onto the surface of the scaffold via a motorized syringe. An image was taken after 5 s, followed by the determination of the contact angle value.

### 2.7 Cytocompatibility of PCL Fibrillar Scaffolds Containing HAn in Preosteoblast Culture

#### 2.7.1 Scaffolds’ Preparation

PCL scaffolds were synthesized for 60 min on sterilized 13 mm diameter glass coverslips (Perfecta, São Paulo, SP, Brazil) adhered with 2 μL of deionized water at a flat aluminum collector. Only coverslips that were uniformly covered by nanofibers were submitted to the experiments. After the residual solvent was evaporated in a desiccator containing silica for 72 h, the samples were individually placed in compartments of 24-well sterile plates. Then, the samples were subjected to the disinfection protocol (washing with 500 μL per sample of 50% ethanol for 10 min, three washes with 500 μL per sample of 70% ethanol for 20 min, washing with 1 mL of sterile phosphate-buffered saline solution (PBS 1×) for 10 min).

#### 2.7.2 Primary Culture of Mouse Calvaria Preosteoblasts

After approval by the Animal Research Ethics Committee of the Sao Paulo State University School of Dentistry-Araraquara (CEUA 04/2020), mouse preosteoblasts (Swiss) were isolated from Calvaria.^18^ Calvaria cells from a 2–3-day old mouse were isolated by enzymatic digestion using solutions of EDTA (Sigma-Aldrich) and collagenase type II (Worthington Biochemical Corp, Lakewood, NJ).

#### 2.7.3 Cell Culture

After isolating the preosteoblasts, they were seeded (3 × 10^4^ cells/sample) in α-MEM culture medium (Gibco, Carlsbad, CA) containing 1% penicillin and streptomycin (Gibco) and 10% fetal bovine serum containing (FBS) and maintained in an incubator at 37 °C and 5% CO_2_ (Isotemp Fisher Scientific, Pittsburgh, PA). The culture medium was replaced every 2 days by means of differentiation (α-MEM containing 10% FBS, 5 M β-glycerophosphate, and 0.2 M l-ascorbic acid) until the time of analysis.

#### 2.7.4 Cell Viability (7, 14, and 21 Days)—AlamarBlue

Cell viability analysis was performed at 7, 14, and 21 days using AlamarBlue reagent (Invitrogen, Carlsbad, CA). 300 μL of DMEM without FBS containing 10% AlamarBlue solution was added to the cells. The cells were incubated (37 °C and 5% CO_2_) in contact with the solution for a period of 4 h when the fluorescence of the samples was determined in a fluorimeter (Synergy H1) at wavelengths of 570 and 600 nm.^19^ The mean fluorescence of the PCL group (control) was considered as 100% cell viability.

#### 2.7.5 Collagen Synthesis Assessment (7, 14, and 21 Days)—Sirius Red

Total collagen synthesis was determined by the colorimetric method of Sirius Red. During 14 and 21 days, the culture medium of each collected sample was stored at −20 °C. For quantification of soluble collagen in the sample, 400 μL aliquots were added to 1.5 mL tubes together with 400 μL of Direct Red solution (Sigma-Aldrich). The samples were incubated at room temperature under agitation at 400 rpm for 1 h. After incubation, the samples were centrifuged (12,000 rpm, 10 min, 4 °C), the supernatant material was discarded, and the pellet was washed with 500 μL of hydrochloric acid (HCl–0.1 M). Another centrifugation was performed, and the supernatant was discarded again. The pellet formed was resuspended in 250 μL of sodium hydroxide (NaOH, 0.5 M). Then, two aliquots of 100 μL of each sample were transferred to a 96-well plate and analyzed in a spectrophotometer at 555 nm (Synergy H1). The average absorbance of the PCL group (control) was considered as 100% total soluble collagen.

#### 2.7.6 Total Protein Production (7 and 14 Days)

Total protein production was determined by the Lowry method.^20^ After 7 and 14 days in culture, cells were lysed with 150 μL of 1% sodium lauryl sulfate (Sigma-Aldrich) for 40 min at room temperature. Then, 100 μL of Lowry Reagent (Sigma-Aldrich) was added, followed by a new incubation for 20 min at room temperature. Finally, 50 μL of Folin and Ciocautel’s Reagent (Sigma-Aldrich) was added, and after 30 min, the protein concentration of each sample was determined by a spectrophotometer at 655 nm (Synergy H1), according to a standard curve containing predetermined concentrations of albumin.

#### 2.7.7 Assessment of Alkaline Phosphatase Activity (7 and 14 Days)—ALP

The analysis of ALP concentration was performed using an Alkaline Phosphatase Kit (Labtest Diagnóstico S.A., Lagoa Santa, MG, Brazil) at 7 and 14 days. From each sample lysed in 1% sodium lauryl sulfate (Sigma-Aldrich), 50 μL of solution was collected and placed in a tube containing 300 μL of buffer and 50 μL of substrate previously incubated at 37 °C for 2 min, as recommended by the manufacturer. After 10 min of incubation, the color reagent was added. Then, 200 μL aliquots of each sample were transferred to a sterilized 96-well plate and then submitted to analysis in a spectrophotometer at 590 nm (Synergy H1). The enzyme activity of ALP was determined using a standard curve with pre-established concentrations of the enzyme.^20^

#### 2.7.8 Cell Adhesion (7 Days)—Fluorescence Microscopy

Cells were seeded on the scaffolds according to their respective groups, and after 7 days, fluorescence microscopy analysis was performed to verify cell adhesion to the scaffolds. Initially, the cells were washed twice with PBS 1× and then fixed with a 4% paraformaldehyde solution for 10 min. Subsequently, the cells were permeabilized by treating them with 0.1% Triton X-100 for 10 min. After additional washes with PBS 1×, the cells were treated with the fluorophore Actin Red (Life Technologies, Eugene, OR) for 20 min at the concentrations recommended by the manufacturer. Nuclear labeling was performed using the DNA marker (Hoechst, Invitrogen), prepared at a concentration of 1:5000 in PBS 1×. Finally, the cells on the scaffolds were analyzed by using a fluorescence microscope (Leica DMI4000B, Leica Microsystems, Wetzlar, Germany) at a magnification of 20×.

#### 2.7.9 Mineralization Nodules (14 and 21 Days)—Alizarin Red

The formation of mineralization nodules was analyzed by the Alizarin Red test in the periods of 14 and 21 days.^20^ Cells were fixed with 500 μL of 70% ethanol for 1 h at 4 °C, followed by washing with deionized water and application of 500 μL of Alizarin Red solution (40 mM in deionized water, pH 4.2, Sigma-Aldrich). The formation of nodules was evaluated quantitatively by dissolving the nodules in cetylpyridine chloride (10% in PBS; Sigma-Aldrich) and reading the absorbance in a spectrophotometer at 692 nm (Synergy H1).

### 2.8 Statistical Analysis

All experiments were performed twice, in such a way that the “*n*” established for every group represents the sum of both occasions. The structural characterization data of the nanofibers (SEM, EDS, FTIR, and TGA) and cell adhesion analysis were carried out descriptively. The calcium release results were evaluated using confidence intervals. Data were analyzed for their sampling distribution (Shapiro-Wilk) and the homogeneity of variances (Levene). For the analysis of the thickness of the nanofibers, interfibrillar space, and WCA, the analysis of variance test (ANOVA) was used, followed by the Tukey post-test. For the analysis of total protein synthesis, alkaline phosphatase synthesis, and mineralized nodules, the analysis of variance test (two-way ANOVA) was calculated, considering two factors (group and analysis period). For models with repeated measurements (cellular viability and collagen synthesis), the sphericity was evaluated by the Mauchly test. When the null hypothesis of the analysis of variances was rejected, multiple comparisons were calculated with Sidak’s post-tests. For all of the inferences, a significance level of 5% was established.

## 3 Results and Discussion

PCL is a synthetic polymer widely evaluated for guided tissue regeneration; this is because it has numerous advantages such as biocompatibility, low cost, versatility of use and handling, and the possibility of combination with other materials.^15,21^ To improve the physicochemical and biological properties of PCL, several researchers have sought to develop PCL solutions containing bioactive molecules according to their target cells. In the present study, PCL fibrillar scaffolds containing HAn were prepared by the electrospinning method to provide a suitable microenvironment for cell anchoring, which should exchange nutrients and metabolites among them and with the medium.^22^

**Figure 2:**
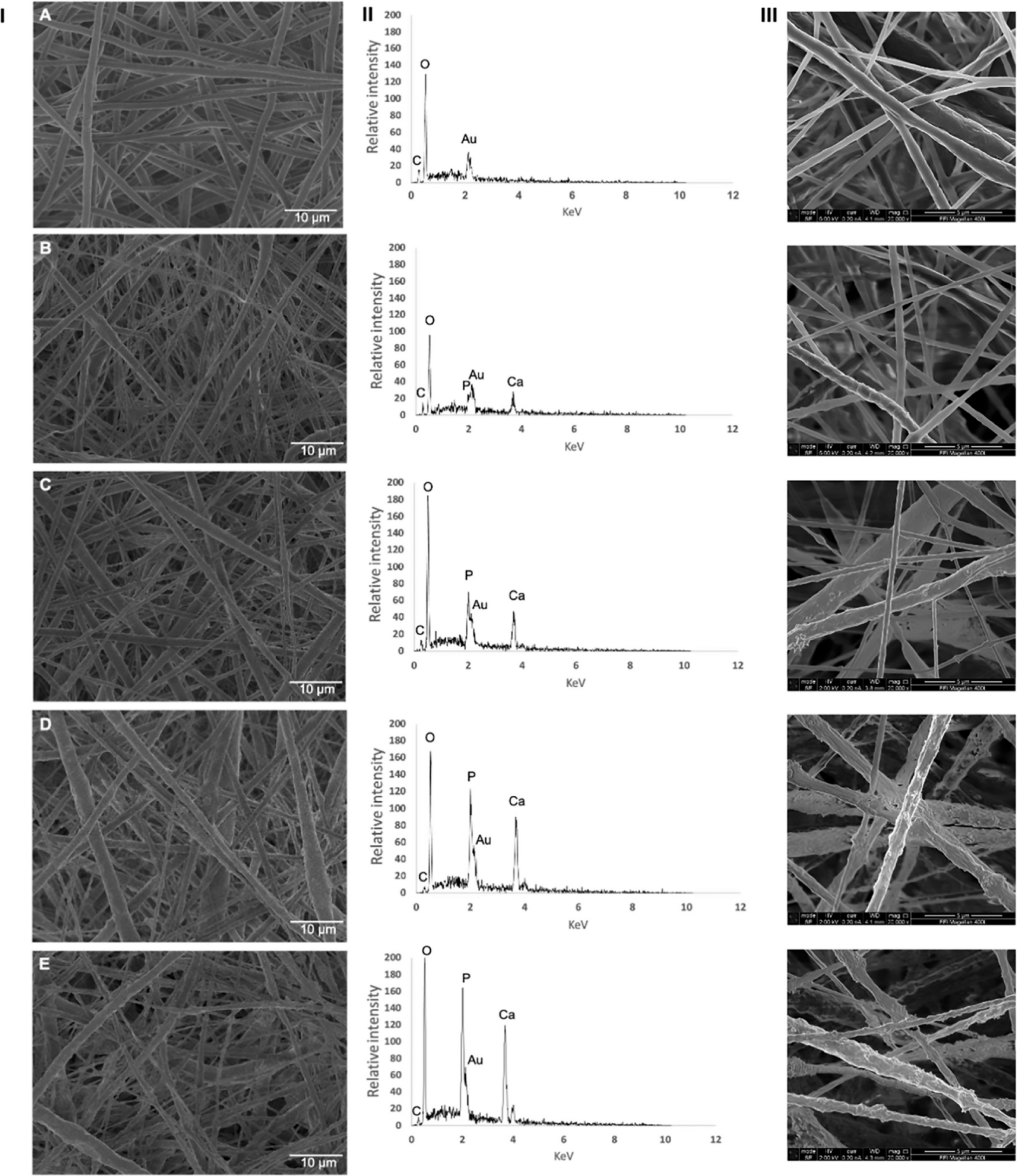
(I) SEM images (2000×), (II) EDS plots, and (III) high-resolution SEM images (20,000×) of (A) the pristine PCL scaffold and the PCL scaffold containing (B) 1% HAn, (C) 2.5% HAn, (D) 5% HAn, and (E) 7% HAn nanofiber formulations. Note the increase in surface irregularities caused by the incorporation of HAn.

**Figure 3:**
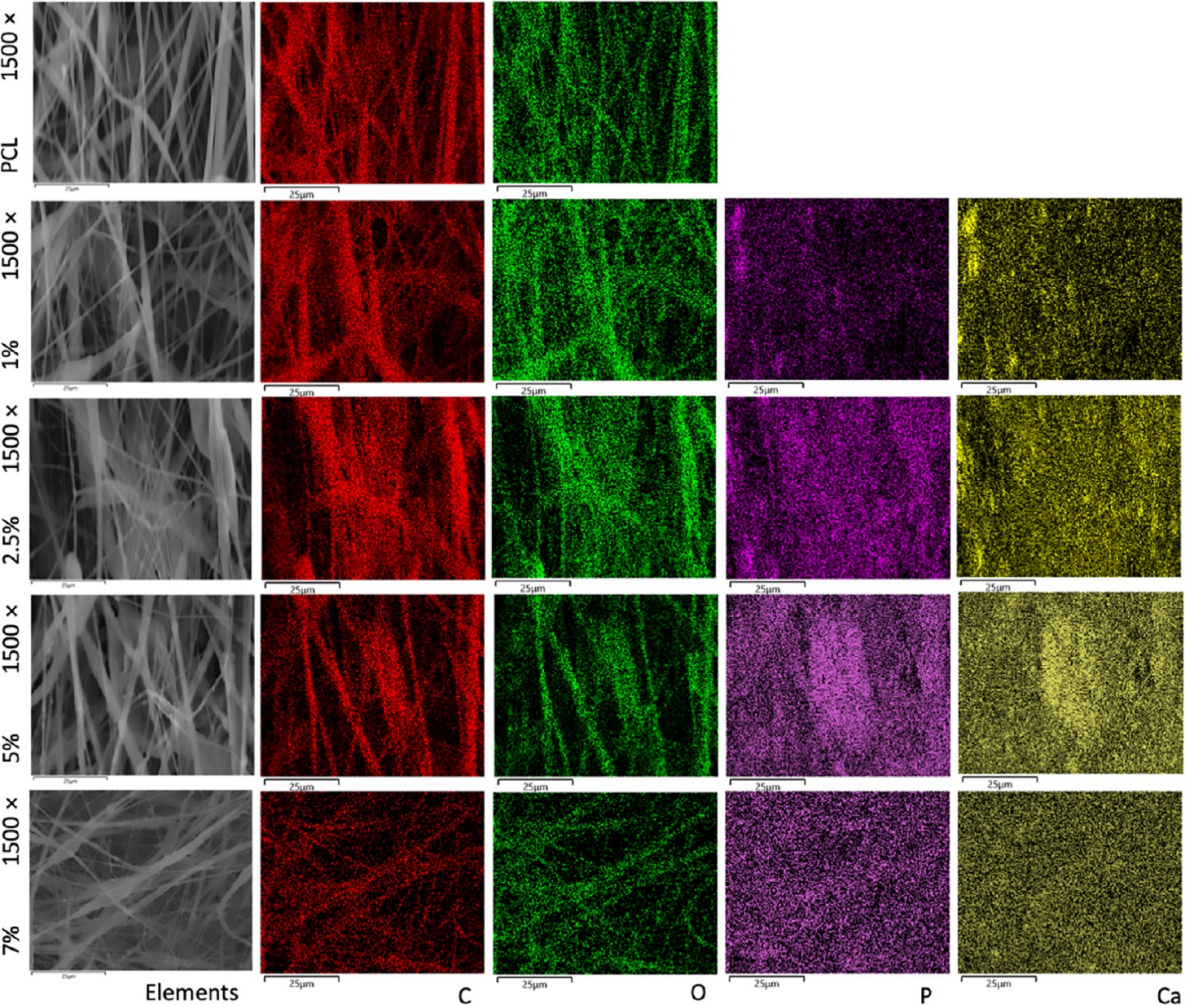
Elemental color map analysis of the pristine PCL scaffold and the PCL scaffold containing 1% HAn, 2.5% HAn, 5% HAn, and 7% HAn nanofiber formulations.

Initially, SEM analysis was performed to determine the morphology, diameter, and distribution of scaffold fibers, which plays a role in the mechanism of tissue regeneration.^15^ The SEM images showed that the pristine PCL scaffold and PCL scaffolds produced at lower concentrations of HAn obtained by the electrospinning technique presented uniform fibers randomly distributed (Figure 2I,III). The EDS plots demonstrate that the greater the incorporation of HAn in the PCL solution, the greater the peaks of P and Ca elements (Figure 2II), without the formation of beads in all tested formulations. The elemental color map analysis revealed the distribution of elements, confirming the uniform presence of HAn within the polymer matrix by the presence of carbon, oxygen, calcium, and phosphorus within the fibers illustrated in Figure 3.

**Figure 4:**
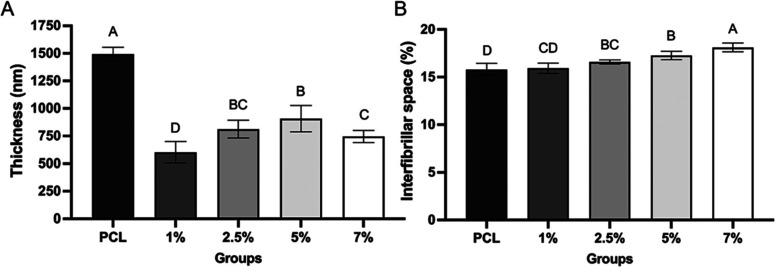
(A) Thickness of fibers (nm), *n* = 240. (B) Interfibrillar space (%), *n* = 8. The columns represent the mean, and the error bars at the top of the columns represent the standard deviation of each group. Equal letters do not differ statistically from each other (ANOVA, Tukey post-test, α = 0.05).

The HAn (mineral phase) incorporated into the fibers made them more irregular and thinner, as shown in Figures 2 and 4. The average value of nanofiber thickness was higher in the PCL group without HAn incorporation, followed by the groups incorporated with 5, 2.5, 7, and 1% HAn (Figure 4A). As for the interfibrillar space, it was directly related to the concentration of HAn incorporated into the PCL solution. Thus, the higher the concentration of HAn in the PCL, the greater the percentage of interfibrillar space (Figure 4B). Previous studies demonstrated that the suspension of different concentrations of nanoparticles, as well as the type of solvent, can modulate the conductivity of the polymeric solution subjected to electrospinning.^23,24^

**Figure 5:**
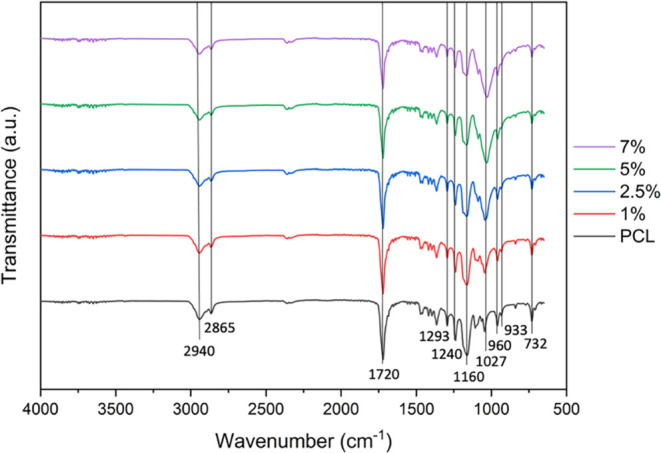
Infrared spectrum (FTIR) of PCL scaffolds containing different concentrations of HAn.

An infrared spectroscopy analysis (Figure 5) showed higher absorption peaks at 2940, 2865, 1720, 1293, 1240, 1160, 1087, 1027, 960, 933, and 732 cm^–1^. The characteristic peaks of PCL are the infrared bands at 2940, 2865, and 1720 cm^–1^, which correspond to symmetrical and asymmetrical CH_2_ bonds, as well as carbonyl bonds (C=O).^25^ The peak at 1293 cm^–1^ corresponds to C–O and C–C bonds, while the peaks at 1240 and 1160 cm^–1^ correspond to symmetric and asymmetric C–O–C bonds, respectively. The bands at 1087, 1027, and 960 cm^–1^ refer to the P–O group, which is characteristic of hydroxyapatite.^26^ Additionally, the presence of inorganic particles delays the decomposition of synthetic biopolymers by acting as a physical barrier.^27^ The slower degradation of this polymer causes new bone tissue to gradually grow through the pores of the biomaterial having more space for trabecular bone growth and enlargement.^28^

**Figure 6:**
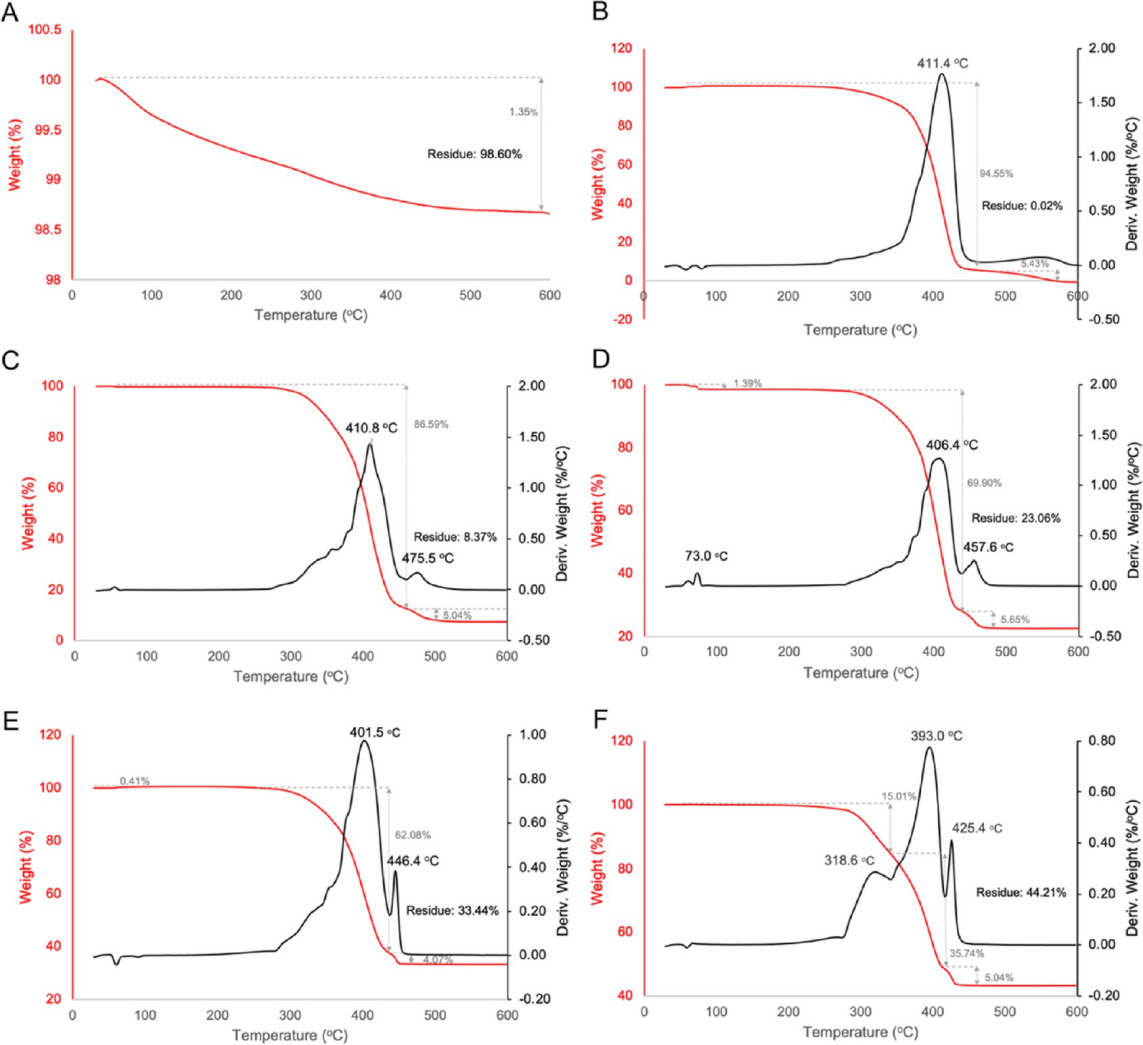
(A) TG curve of HAn. (B) TG/DTG curves of the PCL scaffold. (C) TG/DTG curves of the PCL scaffold made with 1% HAn. (D) TG/DTG curves of the PCL scaffold made with 2.5% HAn. (E) TG/DTG curves of the PCL scaffold made with 5% HAn. (F) TG/DTG curves of the PCL scaffold made with 7% HAn.

Thermal degradation analyses were performed with the aim of trying to better understand the nature of the modification of PCL scaffolds containing different concentrations of HAn (Figure 6). For the HAn, the weight loss of 1.4% in the temperature range of 30–600 °C was attributed to the evaporation of residual water in the sample (Figure 6A). The TGA curve of the pristine PCL scaffold exhibited one decomposition stage (*T*_max_ = 411.4 °C), showing no residual mass at 600 °C (Figure 6B). When different concentrations of HAn were incorporated into the PCL scaffolds, the decomposition of these materials took place in two steps, with the exception of the scaffolds containing 7% HAn, which showed three steps of degradation (Figure 6C–F). The weight loss curves suggest that the thermal stability of the scaffolds decreases with increasing HAn concentration. Likewise, the residual mass formed after heating increases with increasing HAn concentration, indicating the incorporation of HAn into the PCL host. TGA performed in the current investigation also demonstrated a direct relation to HAn incorporation and residue generation (Figure 6C–F), since HAn is a calcium phosphate that shows poor decomposition at the temperature established for the TGA assay (Figure 6A).

**Figure 7:**
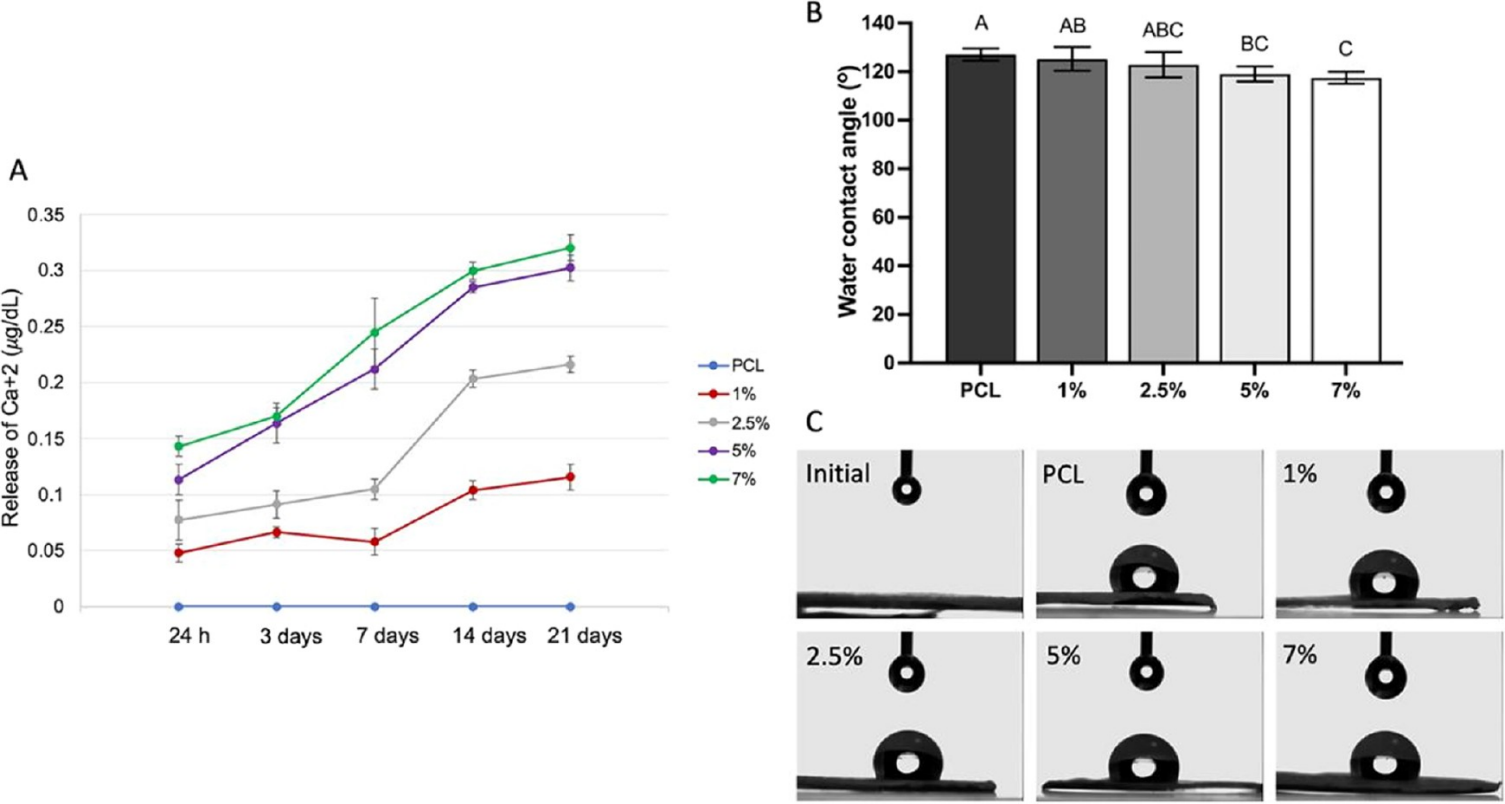
(A) Calcium release (μg/dL), *n* = 6. Points represent mean values per period, and error bars indicate 95% confidence intervals. (B) Water contact angle of the scaffolds. The columns represent the mean, and the error bars represent the standard deviation of each group. Equal letters do not differ statistically from each other (ANOVA, Tukey post-test, *n* = 6, α = 0.05). (C) Image of a water drop on scaffolds (6.5× magnification).

Dwivedi et al.^15^ reported that the slow degradation of PCL (about 2–4 years) may be enhanced after HAn incorporation. Therefore, the increased PCL degradation may favor the osteogenic behavior of bone cells with time, since this polymeric material exhibits space for bone deposition within it. The presence of HAn in the nanofibers favored Ca^2+^ release, being this effect concentration-dependent (the greater the amount of HAn, the greater the release of Ca^2+^). The peak of Ca^2+^ release in 1 and 2.5% groups occurred between 7 and 14 days, while for the 5 and 7% groups, the peak was observed between 3 and 14 days (Figure 7A).

The hydrophilicity analysis showed that the WCA of the PCL scaffold is approximately 130°, indicating a highly hydrophobic surface with low wettability (Figure 7B,C). With the addition of nHA at concentrations of 5 and 7%, the WCA decreased, demonstrating that incorporating nHA into PCL scaffolds can reduce hydrophobicity. The chemical structure of HAn is Ca_5_(PO_4_)_3_(OH), and the presence of hydroxyl (OH) groups likely contributes to the reduction in WCA.^9^ One may suggest that the small Ca^2+^ release into the aqueous medium occurs due to the low degradation rate of the polymer scaffolds and the trapping of HAn inside their fibers. Rezwan et al.^29^ showed that PCL degradation also released acidic byproducts, which can reduce cell proliferation. However, the slight alkalinity of HAn incorporated into the scaffolds may reduce the local acidity, preventing cell damage.^29,30^

**Figure 8:**
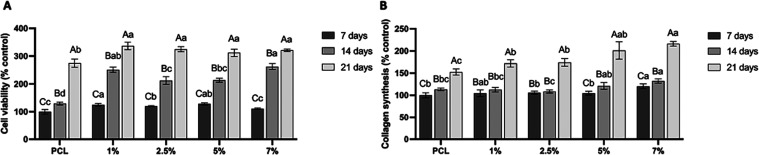
(A) Cell viability (%) and (B) total collagen synthesis (%) of preosteoblasts at 7, 14, and 21 days. The mean fluorescence of the PCL group (control) was considered as 100% cell viability. The columns represent the mean, and the error bars represent the standard deviation of each group. Capital letters compare the groups in the different periods. Lowercase letters compare the different groups within each period. Equal letters do not differ statistically from each other (two-way ANOVA with repeated measures, Sidak post-test, *n* = 8, α = 0.05).

In the present study, the addition of all concentrations of HAn in the scaffolds did not interfere with the cell viability compared to the control group (PCL) and stimulated cell proliferation over time (Figure 8A). Two-way ANOVA showed that cell viability (Figure 8A) is influenced by the concentration of HAn (*F* = 13.036; *p* < 0.001), evaluation period (*F* = 4425.728; *p* < 0.001), and the interaction between HAn concentration and evaluation period (*F* = 21.986; *p* < 0.001). Comparing the effects of interaction, the Sidak post-test showed that in all groups, the longer the analysis period, the higher the cell viability (*p* < 0.05). In the 7-day period of analysis, greater cell viability was observed in the groups where 1% and 5% HAn was incorporated into the PCL solution compared to the other groups (*p* < 0.05). In the period of 14 days, the groups with PCL containing 1 and 7% HAn showed higher cell viability when compared to the other groups (*p* < 0.05). In the 21-day period of analysis, all groups with HAn incorporation showed statistically similar cell viability (*p* > 0.05), which was superior to the PCL group (*p* < 0.05). Despite these interesting data, previous investigations demonstrated controversial results, which seems to be dependent on the cell type used.^3,31,32^

Two-way ANOVA showed that collagen synthesis (Figure 8B) is influenced by the concentration of HAn (*F* = 90.702; *p* < 0.001), evaluation period (*F* = 1373.171; *p* < 0.001), and interaction between the concentration of HAn and the evaluation period (*F* = 13.515; *p* < 0.001). Comparing the effects of interaction, the Sidak post-test showed that in all groups, the longer the analysis period, the greater the collagen synthesis, except in the groups with 1 and 2.5% HAn incorporation. In these two groups, there was no statistical difference between the periods of 7 and 14 days (*p* = 0.159 and 0.323, respectively). Comparing the groups in the different periods, the incorporation of 7% HAn was the one that presented the highest values of collagen synthesis compared to the other groups in all analysis periods (*p* < 0.05).

**Figure 9:**
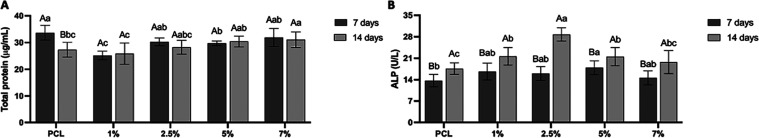
(A) Total protein production (μg/mL) and (B) alkaline phosphatase activity (U/L) of preosteoblasts at 7 and 14 days. The columns represent the mean, and the error bars represent the standard deviation of each group. Capital letters compare each group in the different periods. Lowercase letters compare the different groups within each period. Equal letters do not differ statistically from each other (two-way ANOVA, Sidak post-test, *n* = 8, α = 0.05).

Although the groups with 5 and 7% HAn incorporation showed the highest values of total protein production (Figure 9A), the 2.5% HAn group exhibited the greatest ALP synthesis in comparison with the other groups (Figure 9B). Two-way ANOVA showed that for the production of TP (Figure 9A) there is an effect of the concentration of HAn (*F* = 11.829; *p* < 0.001), the evaluation period (*F* = 7.778; *p* < 0.007), and the interaction between concentration of HAn and evaluation period (*F* = 5.394; *p* < 0.001). Comparing the interaction effects, the Sidak post-test showed that TP production decreased only in the 1% HAn group in the 7-day period. The higher the concentration of HAn, the greater the increase in TP in the 14-day period. For ALP production (Figure 9B), the two-way ANOVA showed that ALP was influenced by the concentration of HAn incorporated into the PCL (*F* = 16.021; *p* < 0.001), evaluation period (*F* = 111.640; *p* < 0.001), and the interaction between HAn concentration and evaluation period (*F* = 8.606; *p* < 0.001). Comparing the interaction effects, the Sidak post-test showed that ALP production was higher in the 14-day period and that the concentration of HAn also affected ALP production. Among all concentrations tested, 2.5% HAn showed the greatest increase in ALP production in the 14-day period.

**Figure 10:**
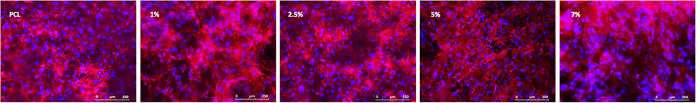
Fluorescence microscopy of osteoblasts cultured on the pristine PCL scaffold and the PCL scaffold containing 1% HAn, 2.5% HAn, 5% HAn, and 7% HAn nanofiber formulations. The cell nucleus was stained in blue, and the cytoplasmic matrix was stained in red. Direct fluorescence microscopy (20× magnification).

The ALP enzyme is highly expressed in mineralized tissue cells and plays a critical role in hard tissue formation. This enzyme increases the local levels of inorganic phosphate, facilitates mineralization, and reduces the extracellular concentration of pyrophosphate, which is an inhibitor of mineral formation.^33^ Biomaterials based on calcium phosphate nanoparticles, such as HAn, release calcium and phosphate ions, promote protein adsorption, and stimulate bone cell differentiation associated with the synthesis of a collagen-rich matrix,^34,35^ as observed in fluorescence microscopy images (Figure 10). Images of cell adhesion on the scaffolds demonstrated no significant differences in the adhesion patterns of cells to scaffolds (Figure 10). The fibrillar scaffolds allowed cell permeation into their structure, which was evident from the difficulty in focusing on certain regions of the images.

**Figure 11:**
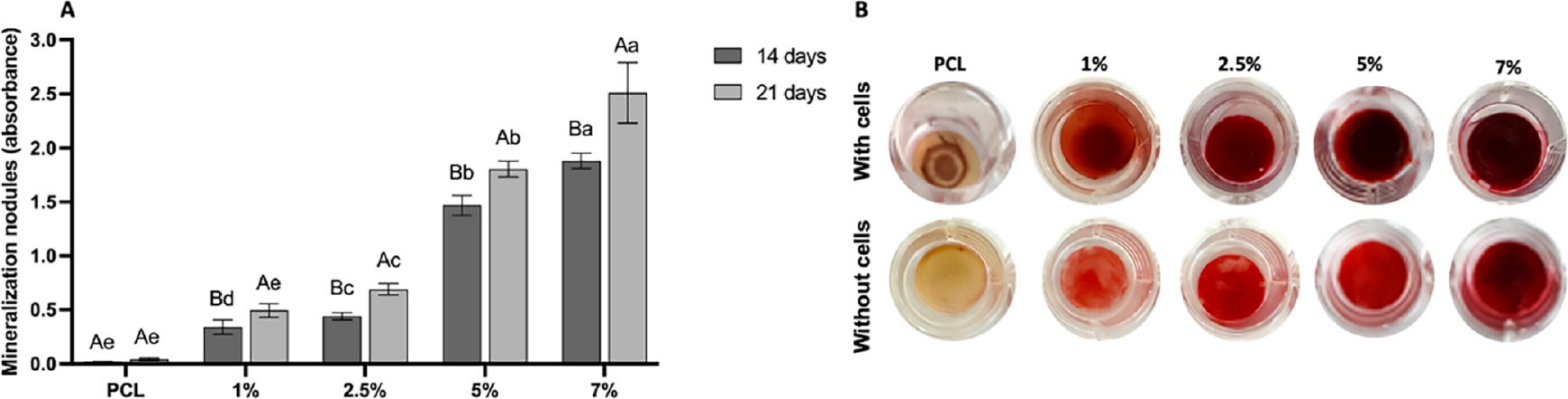
(A) Production of mineralized nodules (absorbance) of preosteoblasts at 14 and 21 days. The columns represent the mean, while the error bars represent the standard deviation of each group. Capital letters compare each group in the different periods. Lowercase letters compare the different groups within each period. Equal letters do not differ statistically from each other (two-way ANOVA, Sidak post-test, *n* = 8, α = 0.05). (B) Images of the scaffolds stained with Alizarin Red after 14 days with and without seeded cells.

At the end of the osteogenesis process, in which bone matrix proteins and extracellular calcium phosphate are deposited, the mineralization of the extracellular matrix takes place.^36^ In the present study, enhanced mineralization nodule formation occurred in those groups where the cells were seeded onto scaffolds containing HAn (Figure 11). Two-way ANOVA showed that the production of mineralized nodules (Figure 11) was influenced by the concentration of HAn present in the PCL solution (*F* = 1195.745; *p* < 0.001), evaluation period (*F* = 139.031; *p* < 0.001), and the interaction between HAn concentration and evaluation period (*F* = 18.876; *p* < 0.001). Comparing the effects of the interaction, the Sidak post-test showed that in all groups, the longer the period of analysis, the higher the production of mineralized nodules, except in the isolated PCL group, in which there was no statistical difference between 14 and 21 days (*p* = 0.705). It was also observed that the greater the amount of HAn incorporated in the nanofibers, the greater the production of mineralized nodules (*p* < 0.05). Thus, one may suggest that the presence of HAn incorporated into PCL fibers served as a center of mineral deposition by cells, favoring the mineralization of the extracellular matrix synthesized by osteoblasts.

The promising data obtained in the present study indicate that electrospinning preparation of PCL scaffolds containing HAn results in interesting membranes that stimulate cell proliferation and the formation of a bone matrix around them. However, data obtained from laboratory studies cannot be extrapolated directly to clinical situations.^37^ In this way, further in vitro and in vivo studies to assess the physical–chemical and biological properties of HAn-loaded biomembranes of PCL associated or not with different bioactive molecules are needed to obtain safe biomaterials eligible to be clinically used for guided bone regeneration.

## 4 Conclusions

The incorporation of different concentrations of nanoparticles of hydroxyapatite (HAn) in a polycaprolactone (PCL) scaffold enhances the physical–chemical and biological properties of biomembranes generated by electrospinning. The concentration of 2.5% HAn allows for better electrospinning of PCL scaffolds (resulting in more homogeneous fibers compared with higher concentrations of HAn) and also stimulates the synthesis of the enzyme ALP, which is essential for tissue mineralization. The PCL scaffold containing 2.5% HAn is the most promising biomembrane formulation for in vitro osteogenesis.
